# Sleep behavior of infants with infantile hemangioma treated with propranolol—a cohort study

**DOI:** 10.1007/s00431-021-04147-3

**Published:** 2021-06-18

**Authors:** Martin Theiler, Nicole Knöpfel, Susanne von der Heydt, Agnes Schwieger-Briel, Isabelle Luchsinger, Alexandra Smith, Kristin Kernland-Lang, Regula Waelchli, Kathrin Neuhaus, Malcolm Kohler, Ralph Gnannt, Sarah F. Schoch, Lisa Weibel, Salome Kurth

**Affiliations:** 1 Pediatric Skin Center, Dermatology Department, University Children’s Hospital Zurich, Steinwiesstrasse 75, CH-8032 Zurich, Switzerland; 2 Vascular Anomalies Board Zurich, University Children’s Hospital Zurich, Zurich, Switzerland; 3 Children’s Research Center, University Children’s Hospital Zurich, Zurich, Switzerland; 4 Department of Pediatric Surgery, Charité University Medicine, Virchow Medical Center, Berlin, Germany; 5 Division of Pediatric Dermatology, Kantonsspital Winterthur, Winterthur, Switzerland; 6 Division of Pediatric Dermatology, Kantonsspital Baden, Baden, Switzerland; 7 Pediatric Skin Center, Division of Plastic and Reconstructive Surgery, University Children’s Hospital Zurich, Zurich, Switzerland; 8 Department of Pulmonology, University Hospital Zurich, Zurich, Switzerland; 9 Division of Pediatric Interventional Radiology, Department of Diagnostic Imaging, University Children’s Hospital Zurich, Zurich, Switzerland; 10 Center of Competence Sleep & Health Zurich, University of Zurich, Zurich, Switzerland; 11 Department of Psychology, University of Fribourg, 1700 Fribourg, Switzerland

**Keywords:** Sleep, Propranolol, Infantile hemangioma, Actigraphy

## Abstract

Sleep problems are frequently reported in infants treated with propranolol for infantile hemangiomas, possibly serving as a marker for a negative impact on central nervous system function. In this cohort study, we objectively investigate the sleep behavior of infants with infantile hemangiomas on propranolol compared to a healthy, untreated control group. Sleep of propranolol-treated infants and controls was investigated using ankle actigraphy and a 24-h diary for 7–10 days at ages 3 and 6 months. The main outcome measures were the *Number of Nighttime Awakenings* and *Sleep Efficiency*. The main secondary outcome measures included *24-hour Total Sleep*, daytime sleep behavior, and parent-rated infant sleep quality and behavioral development based on the Brief Infant Sleep Questionnaire (BISQ) and the age-appropriate Ages-and-Stages Questionnaire (ASQ), respectively. Fifty-four term-born infants were included in each cohort. No group difference in any investigated parameter was seen at age 3 months. At age 6 months, the propranolol group exhibited a decrease in *Sleep Efficiency* and a trend towards an increased *Number of Nighttime Awakenings* compared to the control group. Treated infants at 6 months also had shorter daytime waking periods. *24-hour Total Sleep* was unaffected by propranolol. No negative impact of propranolol on subjective sleep quality and behavioral development was noted.

*Conclusion*: Propranolol exerts a measurable yet mild impact on objectively assessed infants’ sleep measures. Behavioral developmental scores were unaffected. Our results support propranolol as first-line therapy for complicated infantile hemangiomas.

**Table:** 

**What is Known:** *• Sleep disorders are frequently reported in infants with infantile hemangiomas treated with propranolol and often lead to treatment discontinuation.* *• Investigations of the sleep pattern in this patient group using objective measures are lacking.*	
**What is New:** *• The sleep pattern of propranolol-treated infants is assessed using actigraphy and a 24-h sleep diary and compared to healthy, untreated controls.* *• Propranolol leads to a decreased sleep efficiency at night and an increased demand of daytime sleep, yet effects are mild overall.*

**What is Known:**

*• Sleep disorders are frequently reported in infants with infantile hemangiomas treated with propranolol and often lead to treatment discontinuation.*

*• Investigations of the sleep pattern in this patient group using objective measures are lacking.*

**What is New:**

*• The sleep pattern of propranolol-treated infants is assessed using actigraphy and a 24-h sleep diary and compared to healthy, untreated controls.*

*• Propranolol leads to a decreased sleep efficiency at night and an increased demand of daytime sleep, yet effects are mild overall.*

## Introduction

Systemic propranolol is considered first-line treatment for complicated infantile hemangiomas (IH), given its excellent efficacy and safety [1, 2]. As a lipophilic molecule, propranolol can cross the blood-brain barrier, leading to concerns for a potentially negative impact on central nervous system (CNS) function [3, 4]. Indeed, parents have reported sleep disturbances such as insomnia, night restlessness, hypersomnia, and nightmares/night terrors in up to 30% of treated infants [2, 5–8]. Intolerable sleep problems are the most frequent reason for early propranolol discontinuation, which may result in a poorer overall outcome of treated IH [9].

Sleep fulfills an essential function in development and is linked to brain maturation, neural reorganization, and processes of learning and memory [10, 11]. While available data on neurocognitive development of propranolol-treated infants with IH is reassuring [12–15], further investigation of sleep problems and other CNS effects was recommended in a recent review article [6].

Current knowledge on sleep in propranolol-treated infants is mainly based on studies that rely on parental perception of infant’s sleep. Parent reports are a subjective quantification of infant sleep quality, and agreement with objective sleep assessment is poor [16, 17]. Furthermore, parents might be biased by their doctors and information leaflets mentioning sleep problems as a side effect of propranolol therapy. As infant sleep is highly variable and evolving over time, this information might lead parents to interpret normal variability of infant sleep as problematic [18].

Actigraphy is a convenient and cost-efficient method allowing for the recording of sleep data over extended periods in a natural environment, which may be particularly important in infants [19]. Actigraphy has been shown to correlate well with sleep data obtained from polysomnography [20, 21], and its use has significantly increased in pediatric sleep research over the last decades [22, 23]. Combination with a 24-h diary markedly improves the capture of daytime sleep, which is an integral part of total sleep duration in infancy[24].

In this cohort study, the sleep behavior of propranolol-treated infants with IH was objectively investigated through actigraphy and a 24-h diary at ages 3 and 6 months. In addition, subjective sleep parameters and behavioral development were assessed.

## Patients and methods

### Participants

This cohort study consisted of a prospective cohort of infants with propranolol treatment for IH, whose sleep behavior was assessed over time, and a comparison with an identically designed, historical cohort of untreated infants, whose sleep behavior was assessed in the same manner without any temporal matching. The propranolol cohort consisted of otherwise healthy infants treated with propranolol for a complicated IH. The indication for treatment was made independently from and prior to possible inclusion in this project. Infants were recruited prospectively from the vascular anomalies clinics of the University Children’s Hospital Zurich and the Department of Pediatric Surgery, Charité University Medicine, Berlin, from September 2018 through August 2020. The control group was composed of an equal number of untreated matched healthy infants, investigated in an earlier project on infant sleep using the same assessments and study design [10, 24]. Matching criteria included subjects’ sex and chronological age at both assessment timepoints at ages 3 and 6 months (maximum difference of equal or less than ± 10 days at each timepoint). Inclusion criteria were healthy infants aged 0–5.5 months at baseline, born at term (37–42 weeks of gestation), and being mainly breastfed at time of inclusion (at least 50% of daily nutrition intake) to match with the control group [25]. Only vaginal birth was allowed in the control cohort, whereas no restrictions regarding birth mode were applied in the propranolol group. Exclusion criteria included CNS disorders, acute illness, evidence of brain damage, chronic pediatric disease, and a family background of narcolepsy or significant psychiatric disease. Low birth weight (< 2500 g), treatment with medications affecting the sleep-wake cycle (apart from propranolol), and travelling across more than one time zone less than 1 week prior to the measurements also led to exclusion.

Propranolol treatment was performed according to current guidelines [1, 26]. In case of intolerable side effects, patients were offered off-label treatment with atenolol, a hydrophilic cardio-selective beta-blocker (1 mg/kg/day, single daily dose) [27].

Ethical approval was obtained according to local standards (Cantonal ethics committee, BASEC 2018-01366, and 2016-00730), and study procedures were consistent with the declaration of Helsinki. Written parental consent was obtained in all subjects before data collection.

### Study procedures

Assessments were scheduled at ages 3 and 6 months, within a 1-month window centered around the target age. For subjects initiating propranolol treatment after age 3 months, only one assessment at 6 months was performed. In those infants who stopped propranolol and were switched to atenolol, additional measurements on atenolol were performed within the first 2 weeks after atenolol initiation.

Demographic data and patient characteristics were assessed using patient charts and questionnaire data.

**Fig. 1:**
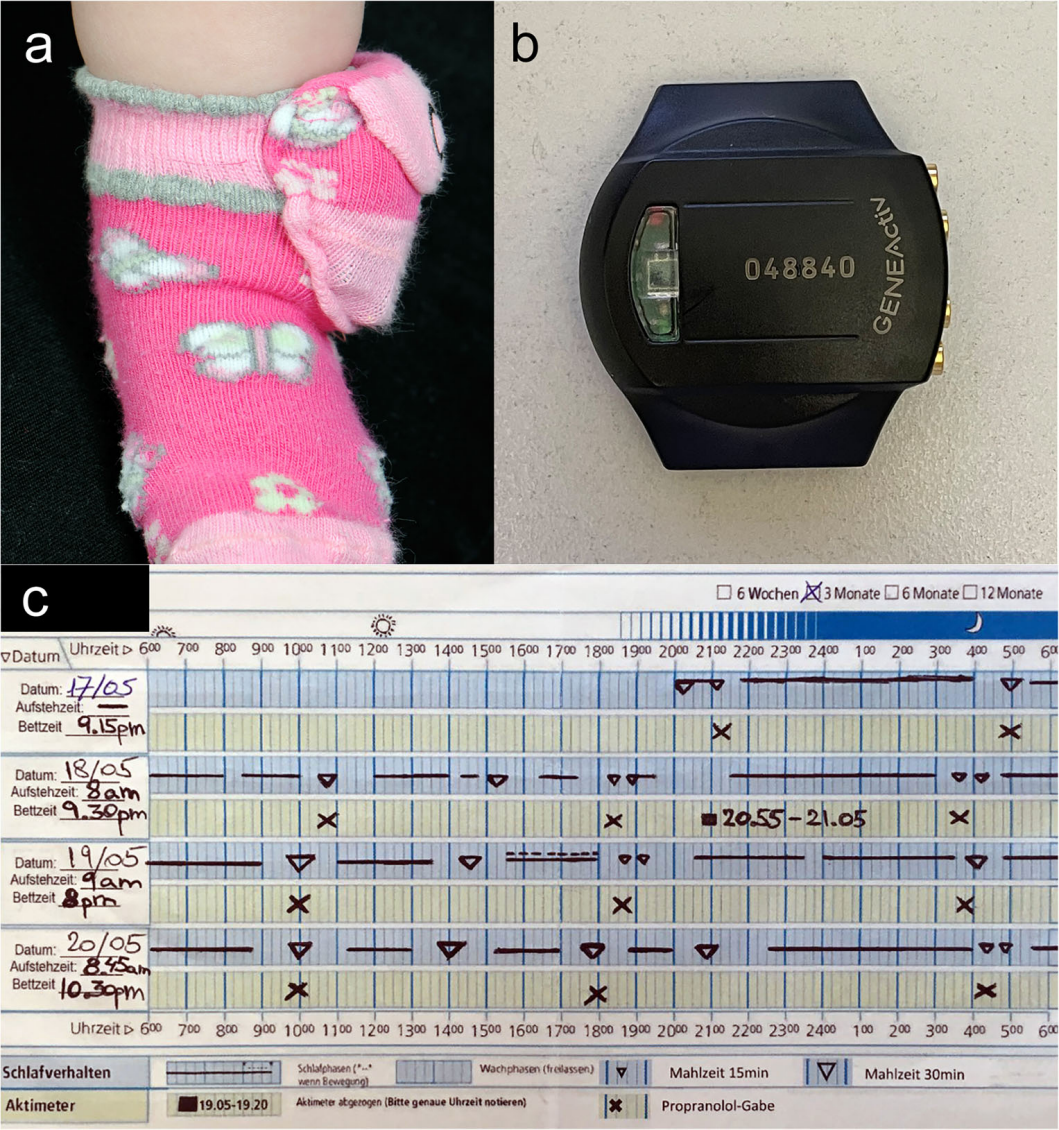
Methods used for assessing objective sleep measures. Attachment (**a**) of the Actigraph (**b**) in a special sock on the left ankle. Representative example of a 24-hour diary (**c**)

Sleep-wake behavior was quantified for 7–10 days at each timepoint, simultaneously acquiring actigraphy and a 24-h diary in the infant’s natural environment (usually at home). GENEActiv movement sensors (Activinsights Ltd, Kimbolton, UK, 43x40x13mm, MEMS sensor, 16 g, 30 Hz Frequency recording resolution) were attached to the infant’s left ankle in a modified sock (Fig. 1).

The 24-h diary was completed in 15-min resolution by caregivers in parallel to the actigraphy recording. Diary data included reporting on infant sleep, external movements occurring during sleep (e.g., sleeping in a stroller/car), feeding episodes, propranolol administration, and bedtimes (clock time) (Fig. 1).

Caregivers in both cohorts completed the Brief Infant Sleep Questionnaire (BISQ) during the measurements, which is a validated survey to assess infant sleep [28]. To investigate the impact of the infants’ sleep behavior on the whole family, both parents’ sleep quality was assessed using the Pittsburgh Sleep Quality Index (PSQI) [29].

The behavioral development of infants in both cohorts was investigated with the parent-completed age-appropriate Ages-and-Stages questionnaire [30].

The Hemangioma Activity Score (HAS) was used to document IH treatment response in the propranolol cohort [31].

### Sleep analysis

Actigraphy data was processed according to in-laboratory standards [10, 24]. A published algorithm was applied to identify infant sleep and wake periods [19]. By applying a previously validated 6-step modification, a better fit with the 24-h diary was achieved [24].

### Primary outcomes

The primary outcomes were the objectively assessed *Number of Awakenings per hour of Night Sleep* and *Sleep Efficiency* (%), which is defined as the ratio of *Total Sleep Time* (at night) and *Sleep Opportunity* (time spent in bed), at 3 and 6 months [10]. *Sleep Efficiency* may be viewed as a marker for overall nighttime sleep quality, as decreased values can correspond to problems falling asleep, increased sleep fragmentation, early waking up, or a combination thereof. These two variables represent a sleep composite that we have previously reported as *Sleep Activity* [10]*.*

### Secondary outcomes

Secondary objective sleep measures included *24-hour Total Sleep* and addressed the four remaining infant sleep composites, which we have previously identified as *Sleep Night*, *Sleep Day*, *Sleep Timing*, and *Sleep Variability* [10]*.* Accordingly, we selected the variables *Sleep Period* (time between *Sleep Onset* and *Sleep Offset* at night in minutes, which represents *Sleep Night*), *Longest Wake* (longest continuous wake period during the day in minutes, which indirectly correlates with the accumulation of daytime sleep need and thus *Sleep Day*), *Sleep Offset* (morning wake time in hours, which represents *Sleep Timing*), and *Variability of Sleep Period* (standard deviation of *Sleep Period* across measurement days (in minutes), which represents *Sleep Variability*).

Secondary outcomes also encompassed subjective data according to the parent-completed questionnaires, including data on infant sleep (*24-hour Total Sleep*, *Number of Nighttime Awakenings*, *Duration of Daytime Sleep*, and *Sleep Problems*), the parents’ sleep quality, and the behavioral development at ages 3 and 6 months.

### Sample size calculation

Determination of the sample size was based on a two-sample t-test power calculation with a two-sided significance level of 0.05 to give greater than or equal to 80% power to detect differences of ≥25% in the *Number of Nighttime Awakenings* in propranolol-treated infants versus controls at 6 months. This resulted in 44 required datasets per group.

### Statistical analysis

Statistical analysis was done using R [32] and RStudio [33] and the packages *ggplot2, MASS*, *mosaic*, *kableExtra*, *pander*, *lme4*, *lmerTest*, *cowplot*, *purr*, *qwraps2*, *psych*, and *dpylr* [34–39]*.* We compared the demographics of the control and propranolol cohort by applying chi-square and t-tests. The efficacy of treatment (baseline, T1, and T2) was assessed using a multilevel model (lmer), with varying intercepts for each patient. Differences in objective and subjective sleep variables, as well as parental sleep quality and behavioral development, were assessed using linear models with group (propranolol/control) as predictors and exact age at measurement start, sex, and gestational age at birth as control variables. Each timepoint was analyzed separately, as we did not have complete datasets for either timepoint, and not enough timepoints to utilize a multilevel model. Alpha level was set to p < 0.05. Because we included many objective and subjective sleep variables, we controlled for multiple comparisons by applying a False Discovery Rate (FDR) correction (Benjamini-Hochberg).

## Results

### Participants

Fifty-four subjects were included in each cohort (detailed patient characteristics are shown in Table 1). There were no significant differences between cohorts except for a lower gestational age in the propranolol group (mean, 39.3 vs. 40.0 weeks, p = 0.001). Thirty-four and 47 datasets were available to calculate objective sleep parameters at 3 and 6 months, respectively. For 7 participants, only 3-month assessments were available due to treatment switch to atenolol before age 6 months (n = 2), vacation in the 6-month measurement window (n = 1), loss to follow-up (n = 2), and withdrawal from the study (n = 2).

**Table 1:** Patient characteristics

	Propranolol cohort (n = 54)	Controls (n = 54)	p = *
Gender			
Male, n (%)	11 (20.4)	11 (20.4)	
Female, n (%)	43 (79.6)	43 (79.6)	
Gestational age at birth (weeks), mean ± SD (range)	39.3 ± 1.2 (37.0–42.0)	40.0 ± 1.0 (36.9–41.4)	**0.001**
Birth weight (grams), mean ± SD (range)	3371 ± 448 (2535–4150)	3373 ± 441 (2580–4600)	0.98
Birth mode			**< 0.001**
Vaginal delivery, n (%)	35 (64.8)	54 (100)	
Cesarean section, n (%)	14 (25.9)	0 (0)	
Unknown, n (%)	5 (9.3)	0 (0)	
Ethnicity			0.16
Caucasian, n	39	52	
Hispanic, n	1	0	
Mixed, n	7	2	
Unknown, n	7	0	
Age of the mother at inclusion (years), mean ± SD (range)	34.0 ± 4.1 (26.0–41.0)	34.1 ± 3.5 (25.0–42.0)	0.93
Age of the father at inclusion (years), mean ± SD (range)	35.7 ± 5.2 (27.0–46.0)	35.8 ± 4.9 (25.0–52.0)	0.90
Co-sleeping rate of infant and parents in the same bed (% of total sleep time)			
At age 3 months, mean ± SD	19.2 ± 28.1	28.9 ± 40.5	0.27
At age 6 months, mean ± SD	24.5 ± 32.7	25.1 ± 34.6	0.93
3-month assessments			
Number of available recordings (male/female)	34 (8/26)	34 (8/26)	
Mean age at initiation (months), mean ± SD (range)	2.9 ± 0.2 (2.4–3.2)	2.9 ± 0.2 (2.4–3.4)	0.80
6-month assessments			
Number of available recordings (male/female)	47 (10/37)	47 (10/37)	
Mean age at initiation (months), mean ± SD (range)	5.8 ± 0.3 (5.5–6.4)	5.8 ± 0.2 (5.5–6.2)	0.48

*Welch two-sample t-test/chi-square test

### Treatment characteristics

**Fig. 2:**
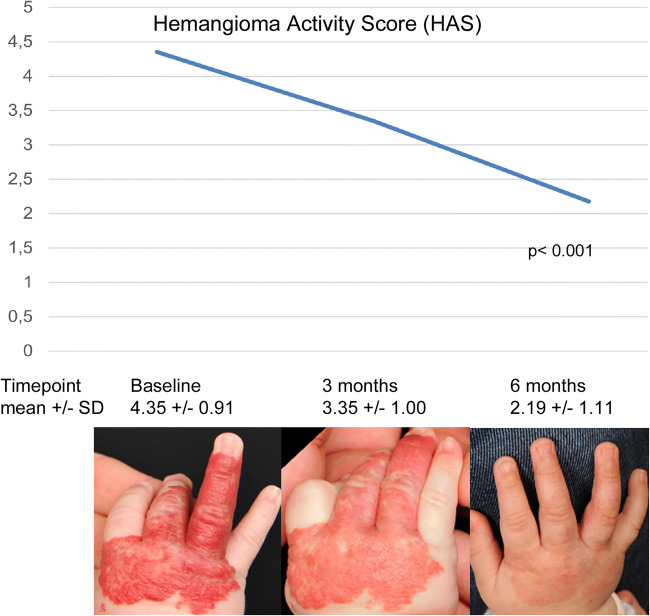
Treatment response of infantile hemangiomas according to the Hemangioma Activity Score (HAS) showing a statistically significant decrease over the study period (p < 0.001)

A total of 70 IH were treated in 54 participants. The mean propranolol dose was 1.9 mg/kg/day at both measurements, and the mean duration of propranolol exposure at the beginning of the measurements was 2.9 and 10.8 weeks at 3 and 6 months, respectively. For details on treatment, please refer to Table 2. Therapy was highly effective, as evidenced by a statistically significant decrease in the HAS across the study period (p < 0.001, Fig. 2). No serious adverse events occurred. Three patients (5.6%) were switched to atenolol at the age of 3 months (n = 2) and 6 months (n = 1), respectively, due to intolerable sleep problems.

**Table 2:** Propranolol treatment characteristics. *IH* infantile hemangioma

Total number of treated IH	70
IH location	
Head/neck, n (%)	47 (67.1)
Trunk, n (%)	13 (18.6)
Extremities, n (%)	10 (14.3)
Reason for treatment	
Life threatening IH, n (%)	0 (0)
Function threatening IH (%)	21 (38.9)
(Risk of) Ulceration (%)	14 (25.9)
Esthetic (%)	34 (63.0)
Age at propranolol initiation (months), mean ± SD (range)	3.2 ± 1.51 (1.1–6.3)
Propranolol dose (mg/kg/day), mean ± SD (range)	
At initiation	2.0 ± 0.3 (0.8–3)
At 3 months	1.9 ± 0.4 (1–3)
At 6 months	1.9 ± 0.3 (1–3)
Duration of propranolol exposure at beginning of 3-month measurements (weeks), mean ± SD (range)	2.9 ± 1.9 (0–5.9)
Duration of propranolol exposure at beginning of 6-month measurements (weeks), mean ± SD (range)	10.8 ± 6.8 (0–20.1)
Hemangioma Activity Score (HAS)	
At initiation ± SD (range)	4.4 ± 0.9 (2–6)
At 3 months ± SD (range)	3.3 ± 1.0 (1.3–5.5)
At 6 months ± SD (range)	2.2 ± 1.1 (0–5)
Serious adverse events, n (%)	0 (0)
Subjects switched to atenolol due to sleep disturbance, n (%)	3 (5.6)

### 3-month objective sleep measures (actigraphy, 24-h diary)

**Fig. 3:**
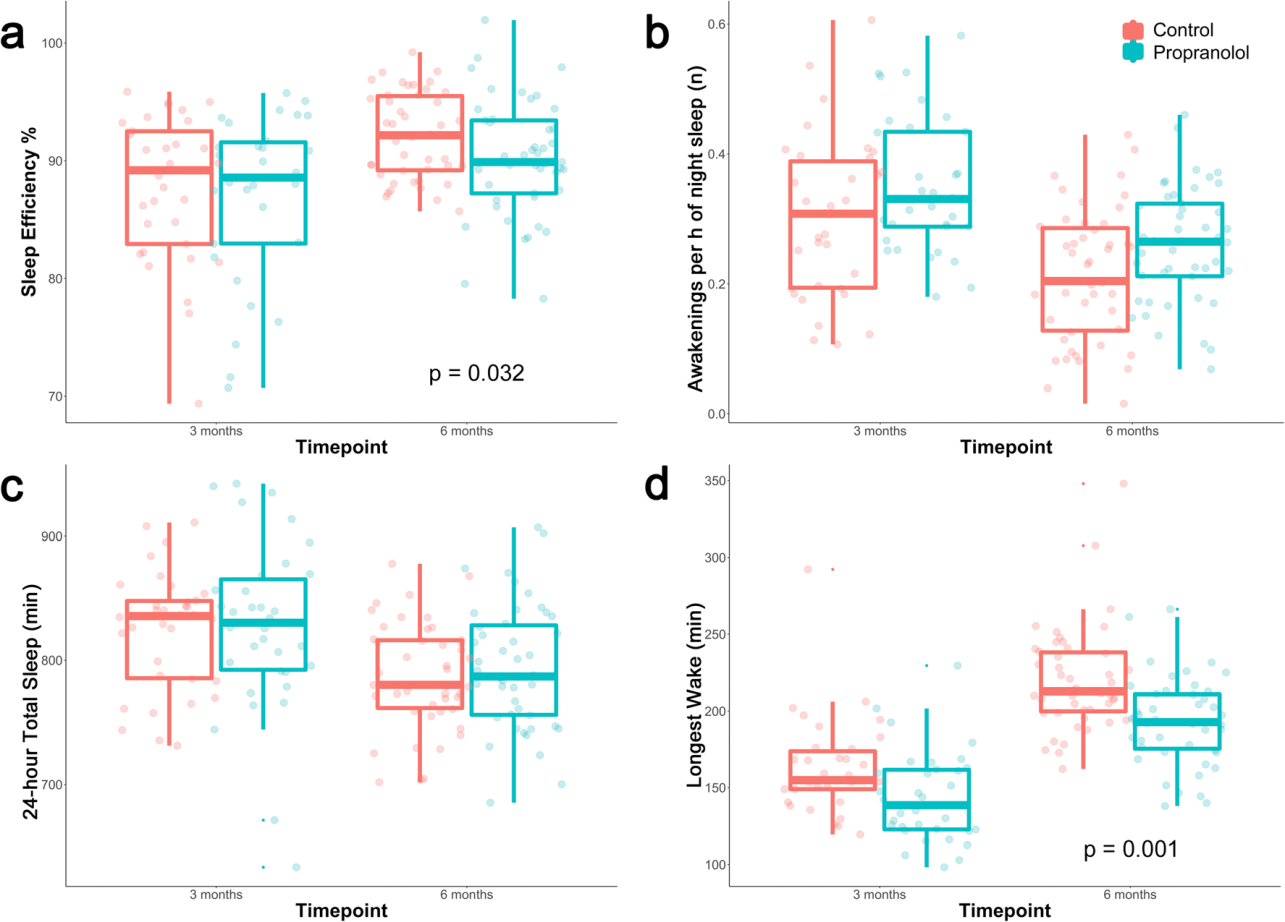
Boxplots of the main objective sleep measures. **a** Sleep Efficiency (%), **b** Number of Awakenings per hour of Night Sleep, **c** 24-hour Total Sleep (min), **d** Longest Wake (min). Sleep Efficiency and Longest Wake showed statistically significant differences between groups at age 6 months

No differences in the objective sleep behavior were detected between the two infant groups (all FDR corrected p-values > 0.05, Table 3). Boxplots displaying objective sleep measures are shown in Fig. 3.

**Table 3:** Objective and subjective sleep measures, behavioral development. *BISQ* Brief Infant Sleep Questionnaire, *PSQI* Pittsburgh Sleep Quality Index, *ASQ* Ages and Stages Questionnaire (age-appropriate)

	3 months				6 months			
	Propranolol	Control	p	Corrected* p	Propranolol	Control	p	Corrected* p
Objective sleep measures (actigraphy, 24-h diary)	**n = 34**				**n = 47**			
Number of Awakenings per hour of Night Sleep, mean ± SD	0.35 ± 0.10	0.30 ± 0.12	0.16	0.25	0.26 ± 0.09	0.21 ± 0.10	**0.018**	0.08
Sleep Efficiency (%), mean ± SD *(Total Night Sleep)/(Total time spent in bed)*	87.0 ± 7.1	87.7 ± 6.2	1.00	1.00	**90.2 ± 5.0**	**92.1 ± 3.6**	**0.005**	**0.032**
24-hour Total Sleep (min), mean ± SD	827.3 ± 68.7	823.9 ± 47.6	0.86	0.92	794.3 ± 49.5	787.3 ± 42.5	0.60	0.70
Sleep Period (min), mean ± SD *(Time between Sleep Onset and Sleep Offset)*	677.3 ± 60.3	645.9 ± 53.1	**0.041**	0.12	657.8 ± 57.4	644.7 ± 42.1	0.21	0.30
Sleep Offset (clock time in h), mean ± SD *(Last minute asleep before Get up Time)*	8.18 ± 1.21	7.84 ± 0.92	0.39	0.50	7.56 ± 0.98	7.22 ± 0.78	0.07	0.14
Variability of Sleep Period (SD), mean ± SD	80.4 ± 33.4	65.2 ± 21.5	0.10	0.17	67.2 ± 25.2	51.8 ± 19.5	0.05	0.13
Longest Wake (min), mean ± SD *(Longest continuous period scored as ‘wake’)*	143.7 ± 29.5	163.3 ± 31.7	**0.036**	0.14	**193.0 ± 28.3**	**219.7 ± 33.5**	**0.0001**	**0.001**
Subjective sleep measures, infants (BISQ)	**n = 29**	**n = 34**			**n = 39**	**n = 47**		
Awakenings during night (n), mean ±SD	2.0 ± 0.3	2.1 ± 0.5	0.90	0.90	2.2 ± 0.6	2.0 ± 0.6	0.31	0.90
24-hour Total Sleep (h), mean ± SD	13.7 ± 2.3	14.2 ± 2.2	0.36	0.90	12.9 ± 1.7	12.8 ± 1.4	0.56	0.90
Daytime Total Sleep (h), mean ± SD	4.6 ± 1.9	4.7 ± 2.1	0.56	0.90	3.1 ± 1.1	3.1 ± 1.1	0.73	0.90
Sleep problems, n (%)	5 (17)	4 (12)	0.76	0.90	13 (33)	14 (30)	0.80	0.90
Problems sleeping through the night	3	3			6	12		
Problems falling asleep	2	1			2	2		
Nightmares	0	0			2	0		
Gastrointestinal complaints	0	0			3	0		
Subjective sleep measures, parents (PSQI)								
PSQI mother (score), mean ± SD	7.0 ± 3.6 (n = 27)	6.7 ± 3.3 (n = 33)	0.64	**-**	6.5 ± 2.8 (n = 37)	5.8 ± 3.1 (n = 47)	0.52	-
PSQI father (score), mean ± SD	4.9 ± 2.9 (n = 17)	5.2 ± 3.2 (n = 32)	0.56	-	4.2 ± 2.7 (n = 22)	5.6 ± 3.1 (n = 45)	0.14	-
Behavioral development (ASQ)	**n = 23**	**n = 34**			**n = 35**	**n = 45**		
Composite ASQ (score), mean ± SD	214.4 ± 40.2	207.9 ± 41.3	0.22	**-**	206.9 ± 41.6	212.4 ± 34.6	0.36	-

*Corrected for multiple comparisons

### 6-month objective sleep measures (actigraphy, 24-h diary)

A significant reduction in infant *Sleep Efficiency* was detected in the propranolol group (mean ±SD: 90.2 ± 5.0 %) vs. the control group at age 6 months (92.1 ± 3.6 %; FDR corrected p = 0.032). In concordance, the *Number of Awakenings per hour of Night Sleep* was increased; this was, however, not significant, when correcting for multiple comparisons (FDR corrected p = 0.08). The *Longest Wake* during the day was significantly shorter, by about 25 min, in treated infants (mean ±SD, 193.0 ± 28.3 min) than controls (219.7 ± 33.5 min; FDR corrected p = 0.001), reflecting an increased demand for daytime sleep in the propranolol group. No differences were evident in the other objective sleep variables, including *24-h Total Sleep* (all FDR corrected p-values >0.05). All results are shown in Table 3 and Fig. 3.

### Subjective sleep measures (BISQ, PSQI)

**Fig. 4:**
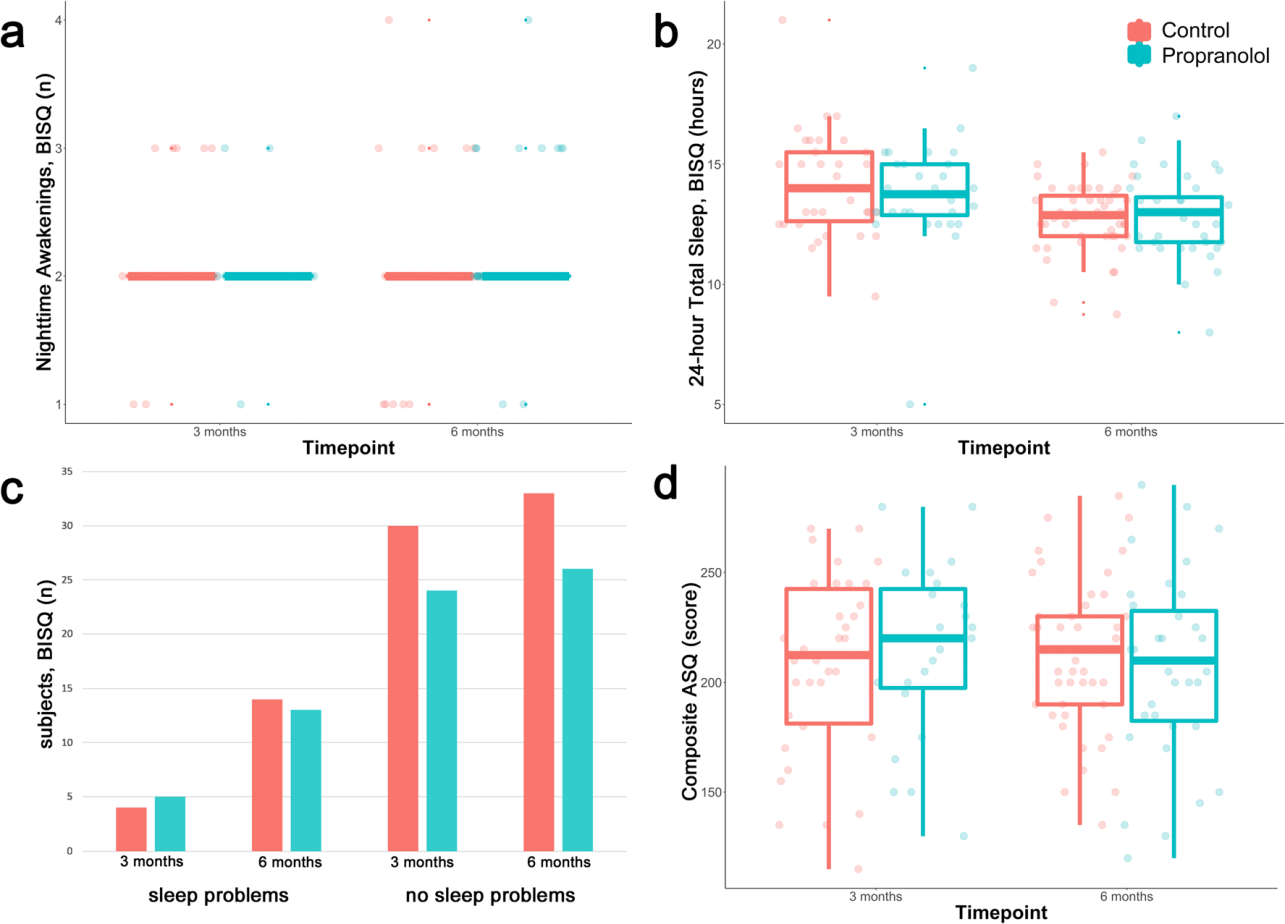
Main results of subjective sleep measures (BISQ) and assessment of behavioral development (ASQ). **a** Number of Nighttime Awakenings, **b** 24-hour Total Sleep (hours), **c** Frequency of sleep problems reported by parents, **d** Scoring on the global ASQ assessing overall behavioral development. No statistically significant differences were found in any of these parameters. BISQ: Brief Infant Sleep Questionnaire. ASQ: Ages-and-Stages Questionnaire

No statistically significant differences occurred in the subjective infant sleep parameters at 3 and 6 months, as demonstrated in Table 3 and Fig. 4 (all FDR corrected p-values > 0.05). About 15% and 30% of parents reported sleep problems in both groups at age 3 and 6 months, respectively. Nightmares (n = 2) and nighttime gastrointestinal complaints (n = 3) were only reported in the propranolol group at 6 months.

Parents’ sleep quality as assessed by means of the PSQI did not differ between groups at either timepoint (p > 0.05).

### Behavioral development (ASQ)

Scoring on the global age-appropriate Ages-and-Stages Questionnaire did not differ between groups at both 3 and 6 months (p > 0.05). For details, see Table 3 and Fig. 4.

### Atenolol treatment

**Fig. 5:**
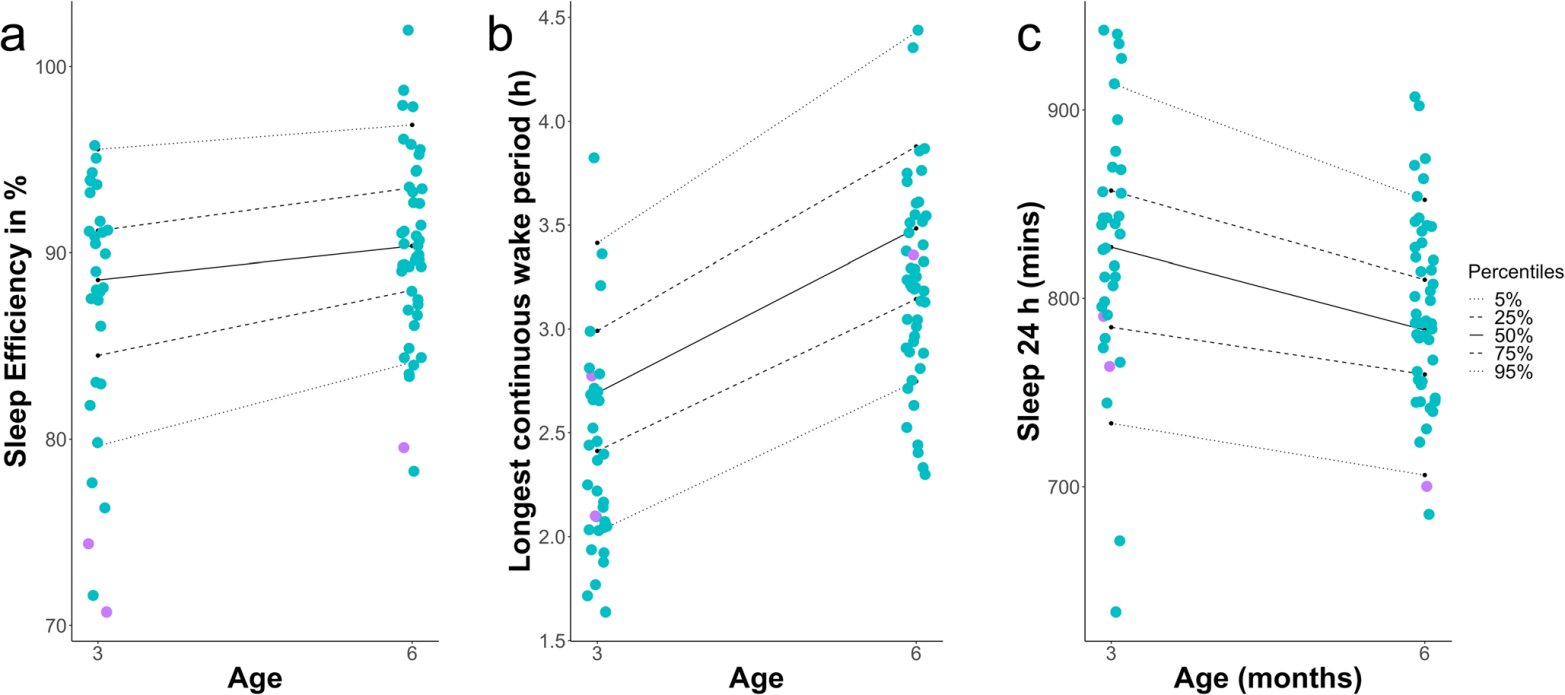
Plots showing the distribution of the objective sleep measurement results of subjects in the propranolol group in relation to the full control cohort (152 infants, percentiles) at 3 and 6 months [10]. Blue dots indicate subjects on propranolol; purple dots (n = 3) indicate subjects on propranolol that had to be switched to atenolol later due to decreased sleep quality. **a** Sleep Efficiency, **b** Longest Continuous Waking Episode, **c** 24-hour Total Sleep

Three patients (2 female/1 male) were switched from propranolol to atenolol therapy due to severe sleep problems. The parents of these 3 infants complained about infants’ difficulties falling asleep and nightly agitation, while one infant additionally experienced nightmares/night terrors. In concordance with these symptoms, all three patients had a relatively low *Sleep Efficiency* with propranolol between 70.7 and 79.5% (Table 4, Fig. 5). Their families reported moderate to considerable improvement of sleep after switching treatment. The low number of subjects precluded statistical analysis. However, descriptively, the *Sleep Efficiency* increased in all three infants (increase ranged from +2.6 to 9.0%). Additionally, the *Longest Wake* period during the day increased by 8.7 to 30.3 min. *24-hour Total Sleep* decreased by 24.3 min and increased by 5.6 and 33.2 min in the three infants, respectively.

**Table 4:** Objective sleep measures in patients switched from propranolol to atenolol (n = 3)

Patient code	Awakenings per hour of night sleep (n)	Sleep efficiency (%)	24-hour total sleep (min)	Sleep Period (min)	Sleep offset (clock time in h)	Variability of **Sleep Period** (SD)	Longest Wake (min)
IHPS24 on propranolol (age 3 months)	0.30	70.7	764.0	788.7	11.1	146.3	166.4
IHPS24 on atenolol	0.33	76.2	739.5	733	11.2	119.3	196.8
IHPS45 on propranolol (age 3 months)	0.52	74.4	790.4	687.7	6.7	83.4	126.0
IHPS45 on atenolol	0.47	77.0	796.0	703	6.1	108.3	141.5
IHPS49 on propranolol (age 6 months)	0.36	79.5	700.2	538.8	8.2	77.4	201.3
IHPS49 on atenolol	0.35	88.6	733.4	619.2	8.8	49.9	210.0

## Discussion

This is the largest study objectively assessing sleep in infants treated with propranolol for IH. Our data show that propranolol exposure mainly affects infants’ nighttime sleep consolidation by reducing *Sleep Efficiency* and, at trend level, increasing the *Number of Nighttime Awakenings*, compared to untreated infants at age 6 months. This is clearly in line with previous reports on sleep disturbance in propranolol-treated infants [2, 4–7] and publications on adult patients reporting increased nighttime awakenings, insomnia, and nightmares while on propranolol and other beta-blockers [40–43]. However, infant *24-hour Total Sleep* was not affected by propranolol. This may suggest a compensatory mechanism, such that decreased *Sleep Efficiency* at night is compensated by increased napping during the day. This concept was indeed evidenced by significantly reduced *Longest Wake* periods in infants receiving propranolol.

While the reduction in *Longest Wake* might be a mere catch-up phenomenon, it could also be due to a direct influence of propranolol on the CNS [3]. We have previously proposed that daytime sleep and *Longest Wake* are markers of general maturation in infancy, based on the observation that they are associated with developmental outcomes of behavior [10].

In contrast to the observations at age 6 months, we did not find any effects of propranolol on sleep behavior at age 3 months. Sleep behavior is continually evolving throughout the first year of life and even beyond—a process of increasing consolidation of the sleep-wake rhythm. Accordingly, *Sleep Efficiency* is expected to be decreased at age 3 compared to age 6 months, as was found in this study. Possible effects of propranolol on *Sleep Efficiency* might, therefore, be obscured. Additionally, capturing sleep behavior using actigraphy is more difficult in younger children, due to less vigorous movements in waking phases. Finally, it may be possible that the effects of propranolol on sleep might be increasing with longer exposure to the medication.

Interestingly, the subjective sleep measures in this study indicated no differences in the infants’ and parents’ sleep quality between groups, except for the occurrence of nightmares in 2 patients (5.1%) in the propranolol group at age 6 months. These results are surprising as they contradict the objective findings of this study and reports from the literature [2, 5], hence supporting previous studies showing that agreement between parent reports and objective sleep measurements is insufficient[16, 17].

While sleep problems in infants may have immediate negative effects on infant-parent bonding and lead to parental stress and depression [44, 45], possible long-term adverse effects on the infant’s behavioral development are of even greater importance [44, 46]. A recent review article reported no significant association of propranolol with CNS effects. However, there seemed to be a trend for increased CNS effects of propranolol compared to other non-corticosteroid treatments or placebo. Of note, propranolol appeared to have a safer profile of CNS effects than corticosteroids, the former mainstay treatment for IH [6].

Current data on behavioral development of children with IH treated with propranolol in infancy are available up to age 7 years and very reassuring [12–15]. In line with this, we did not detect any differences in the behavioral developmental scoring on the Ages-and-Stages questionnaire between treated and untreated children at 3 and 6 months.

Whereas the group differences in *Sleep Efficiency* and *Longest Wake* were statistically significant at infant age 6 months, it may be debated whether these are clinically relevant. If we assume an infant to spend 12 h in bed at night, the mean difference of 1.9% in *Sleep Efficiency* between groups corresponds to an absolute difference of ~14 min in total nighttime sleep, which might be negligible. Taken together with our findings of unaffected *24-hour Total Sleep*, a comparable subjective sleep quality, and normal behavioral development in both groups, we conclude that the impact of propranolol on the sleep behavior of infants, in general, is small. Along these lines, a recent pilot study assessing sleep quality in infants treated with propranolol based on the BISQ and actigraphy in a small proportion of included children did not find a significant impairment of sleep quality and pattern [47].

Nevertheless, a minority of infants seems particularly susceptible to propranolol in terms of sleep, as evidenced by three infants (5.6%) in our study requiring early treatment discontinuation. These infants showed a low *Sleep Efficiency* on propranolol, which improved when they were switched to atenolol. While the number of atenolol-treated infants in this study was low, our findings are in keeping with previous reports suggesting that infants with sleep problems on propranolol might benefit from hydrophilic beta-blockers such as atenolol [27, 48]. Larger-scale studies targeting specifically the small subgroup of infants whose sleep is affected by propranolol are required to understand why high susceptibility towards sleep disturbances under propranolol can in rare cases occur.

How beta-blockers might influence sleep regulation is not yet fully elucidated. Direct action on centrally located beta-adrenergic, noradrenergic, and serotonin receptors, suppression of nighttime melatonin secretion, and even indirect effects via nitric oxide and hydrogen peroxide release have all been postulated [40, 49, 50]. Interestingly, melatonin supplementation was shown to improve sleep in adults treated with beta-blockers [49].

### Limitations

As IH are associated with preterm birth and low birth weight, a significant proportion of propranolol-treated infants had to be excluded [51]. Given the many confounders associated with preterm infants’ sleep behavior (e.g., immature brain development, respiratory problems, hospital admission in a neonatal care unit), only term-born subjects were included in this study. Therefore, we cannot exclude that effects of propranolol on sleep behavior of (previously) preterm infants might be different. In addition, we acknowledge that the used methodological approach, notably the use of historical controls, may cause secular biases and other potential distortion.

### Conclusions

This is the largest study to examine with objective measures whether propranolol treatment of infants with IH is linked to altered sleep behavior. While propranolol therapy was associated with a reduction in *Sleep Efficiency* and an increase in nighttime awakenings at age 6 months, *24-hour Total Sleep* did not differ between treatment and control groups. This observation suggests that infants compensate the effect of propranolol on nighttime sleep throughout the day. Future research should address whether increased napping indeed merely compensates for missed nighttime sleep—or whether the altered daytime sleep in the propranolol group is indicative of potential effects on the CNS and its maturation. No differences in parent-rated infants’ and parents’ subjective sleep quality were detected, and infants on propranolol had normal behavioral development at ages 3 and 6 months. The overall impact of propranolol on infants’ sleep seemed mild.

## Abbreviations

IH: Infantile hemangioma
BISQ: Brief Infant Sleep Questionnaire
ASQ: Ages-and-Stages Questionnaire
PSQI: Pittsburgh Sleep Quality Index
